# Prognostic and immunotherapeutic implications of bilirubin metabolism‐associated genes in lung adenocarcinoma

**DOI:** 10.1111/jcmm.18346

**Published:** 2024-05-02

**Authors:** Kangqi Ren, Xiean Ling, Lin Chen, Zeyao Li, Tonghai Huang

**Affiliations:** ^1^ Department of Thoracic Surgery Shenzhen People's Hospital (The Second Clinical Medical College, Jinan University; The First Affiliated Hospital, Southern University of Science and Technology) Shenzhen China

**Keywords:** bilirubin metabolism, immune therapy, LUAD, prognosis, prognostic signature, risk model

## Abstract

Lung adenocarcinoma (LUAD) is a major subtype of non‐small‐cell lung cancer and accompanies high mortality rates. While the role of bilirubin metabolism in cancer is recognized, its specific impact on LUAD and patient response to immunotherapy needs to be elucidated. This study aimed to develop a prognostic signature of bilirubin metabolism‐associated genes (BMAGs) to predict outcomes and efficacy of immunotherapy in LUAD. We analysed gene expression data from The Cancer Genome Atlas (TCGA) to identify survival‐related BMAGs and construct a prognostic model in LUAD. The prognostic efficacy of our model was corroborated by employing TCGA‐LUAD and five Gene Expression Omnibus datasets, effectively stratifying patients into risk‐defined cohorts with marked disparities in survival. The BMAG signature was indeed an independent prognostic determinant, outperforming established clinical parameters. The low‐risk group exhibited a more favourable response to immunotherapy, highlighted by increased immune checkpoint expression and immune cell infiltration. Further, somatic mutation profiling differentiated the molecular landscapes of the risk categories. Our screening further identified potential drug candidates preferentially targeting the high‐risk group. Our analysis of critical BMAGs showed the tumour‐suppressive role of FBP1, highlighting its suppression in LUAD and its inhibitory effects on tumour proliferation, migration and invasion, in addition to its involvement in cell cycle and apoptosis regulation. These findings introduce a potent BMAG‐based prognostic indicator and offer valuable insights for prognostication and tailored immunotherapy in LUAD.

## 1 INTRODUCTION

Lung cancer (LC) is the primary cause of cancer‐related mortality, representing over 20% of all deaths due to cancer.^1^ Lung adenocarcinoma (LUAD), the primary subtype of non‐small‐cell lung cancer (NSCLC), has attracted significant attention in research in oncology due to its prevalence and complex molecular profile.^2^ Despite advancements in targeted therapies and diagnostics, clinical outcomes remain poor, often attributed to detection in the late‐stage and inherent heterogeneity of LUAD patients.^3^ Recent research has identified the molecular aberrations and mutational patterns hallmark of LUAD, such as driver mutations in EGFR, KRAS, RET, ALK and ROS1, laying the foundation for targeted treatment approaches.^4^, ^5^, ^6^ Nonetheless, patients respond differently to these therapies, highlighting the urgent need for refined biomarkers to improve patient stratification and treatment optimization.

The exploration of LUAD tumour microenvironment (TME) has revealed its critical role in the progression and treatment resistance of the tumour. Within the TME, the dynamic interaction between tumour cells, immune cells and stromal components influences the efficacy of immunotherapies, such as PD‐1/PD‐L1 inhibitors, that have revolutionized the treatment landscape for LUAD.^7^, ^8^ However, variations in patient responses to immunotherapy, with a significant fraction exhibiting resistance or minimal benefit, highlight the necessity to decipher the mechanisms driving this diversity.

With this background, the investigation of metabolic pathways associated with cancer has emerged as a promising avenue for identifying novel therapeutic targets and biomarkers. Traditionally associated with liver function, bilirubin metabolism has attracted attention for its potential role in cancer through its antioxidant, anti‐inflammatory and immunomodulatory properties.^9^ Preliminary studies have suggested an association between bilirubin metabolism and cancer progression, with roles of enzymes such as haem oxygenase‐1 and biliverdin reductase involved in the modulation of the TME and tumour growth.^10^, ^11^ Recent epidemiological evidence highlights observational relationships between bilirubin and LC. One global metabolomic analysis identified bilirubin as a significant biomarker in lung cancer (LC) differentiation.^12^ Subsequent research revealed each 0.1 mg/dL increase in bilirubin leads to nearly a 5% reduction in LC in male smokers; in fact, a large British cohort confirmed an 8% risk reduction per 0.1 mg/dL increase.^13^, ^14^ These findings suggest the potential role of bilirubin as a preventive biomarker against LC. Nonetheless, the precise influence of bilirubin metabolism on the pathogenesis and therapeutic outcomes of LUAD remains poorly understood.

Our study seeks to fill this lacuna by focusing on bilirubin metabolism‐associated genes (BMAGs) and their prognostic value in LUAD. We aim to offer novel insights into their role in LUAD prognosis and immunotherapy response, by constructing a risk prognostic model based on BMAGs. The present research contributes to the burgeoning field of cancer metabolism and promises to refine LUAD treatment strategies, paving the way for personalized therapies that can improve patient outcomes significantly.

## 2 MATERIALS AND METHODS

### 2.1 Data collection and preprocessing

Publicly available LUAD datasets, including TCGA‐LUAD and five Gene Expression Omnibus (GEO) datasets (validation datasets: GSE3141, GSE31210, GSE37745, GSE50081 and GSE68465) were included in our analysis. IMvigor210 and GSE78220 datasets were used to assess the response to immune checkpoint therapy. Data for raw gene expression were preprocessed and normalized using robust multi‐array average and quantile normalization methods. For downstream analyses, clinical information, including patient demographics, tumour stage and survival data, was collected.

### 2.2 Construction of a prognostic risk model

The differentially expressed BMAGs were identified by performing gene set enrichment analysis (GSEA) using the Molecular Signatures Database (MSigDB) curated gene sets. BMAGs differentially expressed significantly between LUAD and adjacent normal tissues were selected for further analyses. The BMAGs implicated in overall survival in TCGA‐LUAD were identified by univariate Cox regression analysis. A risk prognostic model based on the identified prognosis‐related BMAGs was constructed employing Least Absolute Shrinkage and Selection Operator (LASSO) regression analysis. A risk score for each patient was calculated using the linear combination of the gene expression values weighted using the LASSO regression coefficients.

### 2.3 Validation of the prognostic risk model

The performance of the risk prognostic model was evaluated employing Kaplan–Meier survival analysis and time‐dependent receiver operating characteristic (ROC) curves in the training (TCGA‐LUAD) and validation (IMvigor210) cohorts. Patients were stratified into high‐ and low‐risk groups based on the median risk score.

### 2.4 Nomogram construction

The relationship between the identified risk factors (such as the risk score and clinicopathological variables) and overall survival in LUAD patients was visualized by constructing a nomogram. The performance of the nomogram was assessed employing Harrell's concordance index (C‐index) and calibration curves.

### 2.5 Immunotherapy prediction

The IMvigor210 dataset was used to assess the response to immune checkpoint blockade (ICB) in high‐ and low‐risk groups identified by the BMAGs prognostic model. The GSE78220 dataset containing transcriptomic data of pretreatment melanomas and having received ICB therapy was also retrieved to explore the clinical significance of the BMAG risk model in predicting ICB efficacy.

### 2.6 Somatic mutation and tumour mutation burden (TMB) analysis

Somatic mutation data from TCGA‐LUAD were analysed by the TCGA GDC data portal to identify differences in mutation profiles between the high‐ and low‐risk groups. MutSigCV was used to detect significantly mutated genes in each group, and the mutation landscape was visualized by generating OncoPrint plots. The (TMB) of the two risk groups was calculated and its association with overall survival and the risk score was assessed, respectively, using Cox regression analysis and Spearman's rank correlation.

### 2.7 Immune landscape analysis

Immune scores of each prognostic‐related BMAG for the high‐ and low‐risk groups were calculated using ssGSEA. The ESTIMATE algorithm was employed to assess the degree of overall immune cell infiltration. The 22 levels of immune cell infiltration (*n* = 22) and immune checkpoint molecule expressions (*n* = 47) in two risk groups were analysed by employing ssGSEA. The potential clinical benefit of immunotherapy in the two risk groups Tumour Immune Dysfunction and Exclusion (TIDE) algorithm was employed to estimate. In addition, we evaluated the immunotherapy response by detecting three common immunotherapeutic predictive biomarkers, including tumour rejection signature (TRS), cytolytic activity (CYT) score and Th1/interferon‐gamma (IFN‐γ) score. The TRS score was calculated as the geometric mean of the levels of 18 immune rejection‐related gene expressions. The CYT score was determined by averaging the expression levels of two key cytolytic effectors, granzyme A and perforin 1.^15^ Higher CYT scores indicate enhanced immune cell cytolytic activity, which has been correlated with favourable immunotherapy response. The Th1/IFN‐γ score was estimated using the geometric mean of the expression levels of genes, including IFNG, TBX21, STAT1, STAT4, IRF1, CXCL9, CXCL10 and CXCL11.^16^

### 2.8 Prediction of therapeutic drugs

The Cancer Therapeutics Response Portal (CTRP) has a large collection of drug sensitivity data for over 480 small molecules tested across various cancer cell lines.^17^ The PRISM database offers an extensive drug sensitivity dataset, encompassing more than 4500 small molecules screened across a diverse panel of cancer cell lines.^18^ We employed a dual‐pronged approach using CTRP and PRISM‐derived drug response data to identify candidate therapeutic agents with potentially increased drug sensitivity specifically in the high‐risk group. Initially, we examined a differential drug response between two risk groups to identify compounds with lower estimated area under the curve (AUC) values in the high‐risk group. Lower AUC values indicate enhanced drug sensitivity in the high‐risk group. Subsequently, Spearman correlation analysis between the AUC value and risk score was performed to select compounds exhibiting a negative correlation coefficient (Spearman's *r* < −0.30 for CTRP or −0.35 for PRISM).

### 2.9 Cell culture and transfection

Human LC cell lines (including NCI‐H1975 and A549 cells) and normal human bronchial epithelial cell line 16HBE were procured from the American Type Culture Collection. NCI‐H1975 and A549 were grown in Dulbecco's modified Eagle medium (Gibco, USA) with 10% foetal bovine serum (FBS, Gibco) and 1% penicillin–streptomycin (Solarbio, China) in a cell culture incubator at 37°C with 5% CO_2_. Small interfering RNA targeting FBP1 and the relative control (si‐NC) were obtained from RiboBio, and transfection was performed employing Lipofectamine 3000 (Invitrogen) following the manufacturer's instructions.

### 2.10 RNA extraction and quantitative real‐time‐polymerase chain reaction (qRT‐PCR)

The total RNA of cell lines were extracted by TRIzol reagent (Takara). The cDNA was synthesized using PrimeScript™ RT Master Mix (Takara). The level of gene expression was detected by qRT‐PCR employing qPCR SYBR Green Master Mix (Yeasen). Relative expression of the genes was normalized to β‐actin using the 2^−ΔΔCT^ method. The sequences for the PCR primers are provided as follows: FBP1 forward primer: CAGTCGGTCTGTCAGTCCTC FBP1 reverse primer: TGGGGCTCTTCTTGTTAGCG.

### 2.11 Western blotting

Cells were lysed in ice‐cold RIPA (Beyotime Biotechnology) containing phenylmethylsulfonyl fluoride (Solarbio). Cell protein lysates were separated by sodium dodecyl sulfate‐polyacrylamide gel electrophoresis, followed by transfer on to PVDF membranes. Membranes were then blocked with 5% nonfat milk for 1 h and incubated overnight with specific primary antibodies at 4°C. The antibodies used in the study are as follows: fructose bisphosphate 1 (FBP1; 12,842‐1‐AP, Proteintech), B‐cell lymphoma 2 (Bcl‐2; 26,593‐1‐AP, Proteintech), Bcl‐2‐associated X‐protein (Bax; 50,599‐2‐lg, Proteintech), β‐actin (20536‐1‐AP, Proteintech, China), c‐MYC (10828‐1‐AP, Proteintech) and Cyclin‐dependent kinase 4 (CDK4; 11,026‐1‐AP, Proteintech).

### 2.12 Colony formation assays

For colony formation assay, NCI‐H1975 or A549 cells (1000 cells per well) were seeded into 6‐well plates and cultured for 10 days. Then, the cells were fixed with 4% paraformaldehyde and finally stained with 0.5% violet crystal.

### 2.13 Cell migration and invasion assays

The capacity for cell migration and invasion was measured by using Transwell chambers (Corning) with or without Matrigel. LC cells (1 × 10^5^ cells) were transfected with the serum‐free medium and inoculated in the upper chamber, while 600 μL medium supplemented with 15% FBS was added into the bottom chamber. After incubation for 48 h at 37°C, the Transwell chambers were fixed with paraformaldehyde (4%) and stained with crystal violet. The cell migration ability was assessed by counting the cells under a microscope. For the wound healing assay, the tip of a 10 μL pipette was used to create the wound when the cells reached 90%confluence. Cell images were recorded at 0 and 48 h.

### 2.14 Statistical analysis

Experimental data were processed using GraphPad Prism software (V 9.0). Differences between the two groups were estimated using Student's *t*‐test. Statistical significance was determined as *p < 0.05, **p < 0.01, ***p < 0.001.

## 3 RESULTS

### 3.1 Establishment of prognostic signature identified by survival‐related bilirubin metabolism‐associated genes

**FIGURE 1:**
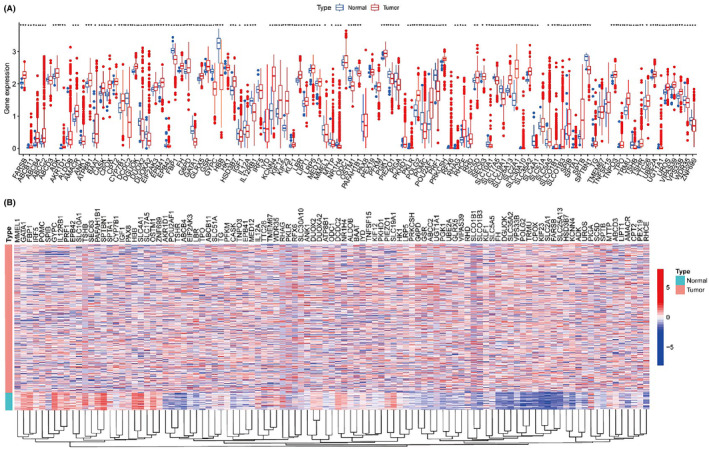
Expression pattern of bilirubin metabolism‐associated genes (BMAGs) in lung adenocarcinoma (LUAD). A: Bot plot of 104 BMAGs in LUAD and normal groups. B: A heatmap of the expression of 104 BMAGs in LUAD and normal groups.

**FIGURE 2:**
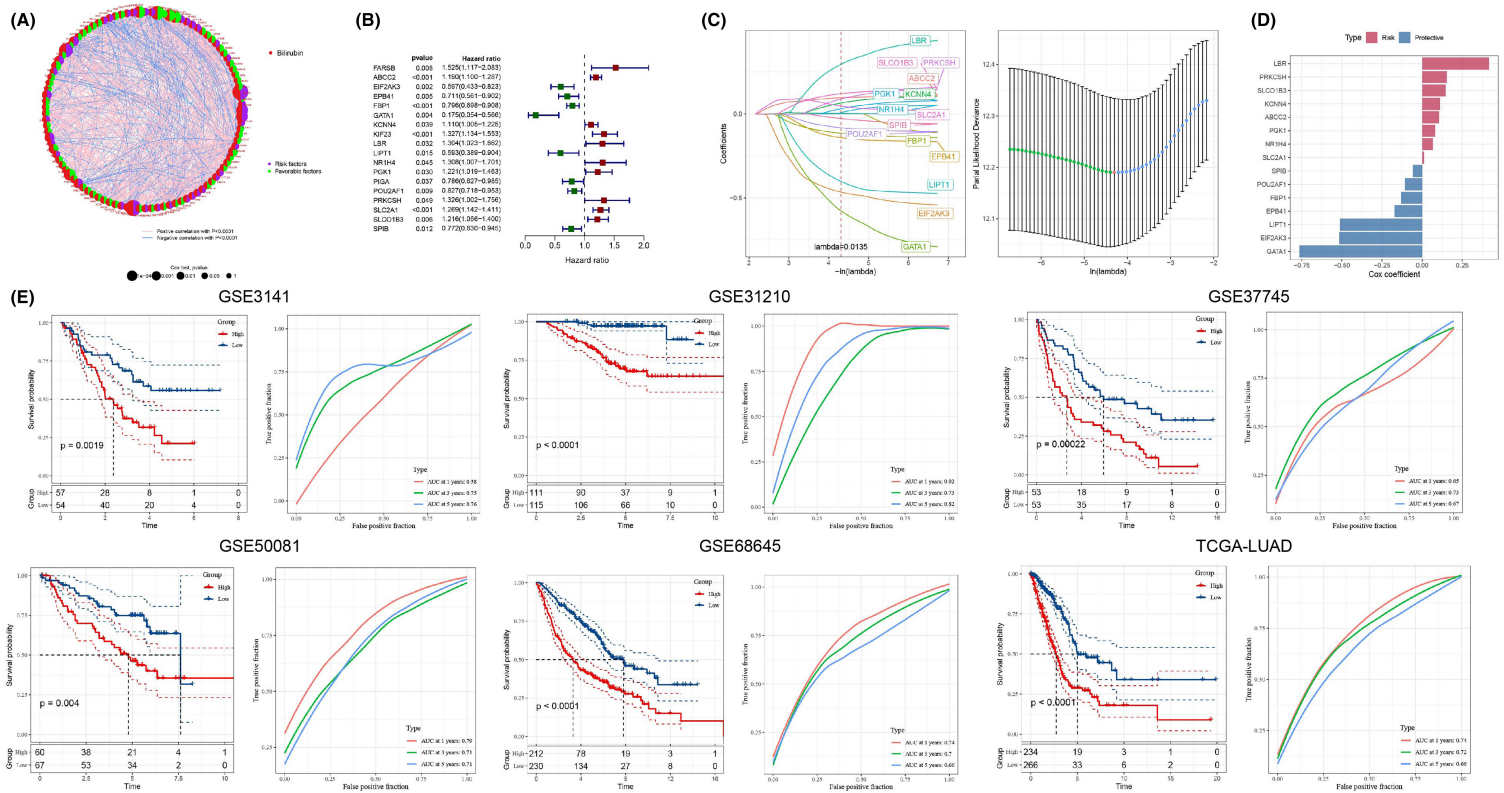
Construction of a bilirubin metabolism‐associated genes (BMAG) prognostic model. A: The correlation network of 104 BMAGs. B: A univariate regression analysis of 18 BMAGs. C: Least Absolute Shrinkage and Selection Operator analysis of 15 BMAGs. D: The multivariate Cox regression coefficients for each BMAG in the risk model. E: Survival curves of BMAG risk model in Gene Expression Omnibus and The Cancer Genome Atlas datasets.

We compiled a set of 104 BMAGs from the GSEA database and observed a significant upregulation of the majority of these BMAGs in LUAD samples (Figure 1A,B). Interestingly, certain genes, including FH, DGUOK, SLC35A2, VPS33B, POLG2, TRMU, CPOX, KIF23, SLC2A1, FARSB, SLC25A13, AKR1D1, POU2AF1, TSHR and ABCB4, were expressed at lower levels in LUAD patients. We also delineated the interaction network among the BMAGs and additional genes (Figure 2A). Subsequently, univariate regression analysis revealed statistically significant correlations of 18 BMAGs with survival outcomes in LUAD (Figure 2B). These 18 BMAGs were also expressed differentially within LUAD contexts (Figure 1A). Through LASSO regression analysis, we identified an optimal subset of 15 BMAGs for prognostic modelling (Figure 2C). The expression of the 15 BMAGs was in File S1. Utilizing these features, we established a prognostic model designating eight BMAGs as risk factors and seven as protective factors in LUAD (Figure 2D). Remarkably, the prognostic signature of the resulting BMAGs represented the best performance in five GSE datasets and the TCGA‐LUAD cohort (Figure 2E). This signature efficaciously categorized patients with LUAD into high‐ and low‐risk groups, providing a novel methodology for distinguishing and potentially reducing risks associated with LUAD.

### 3.2 Efficacy verification of bilirubin metabolism‐associated gene‐prognostic signature

**FIGURE 3:**
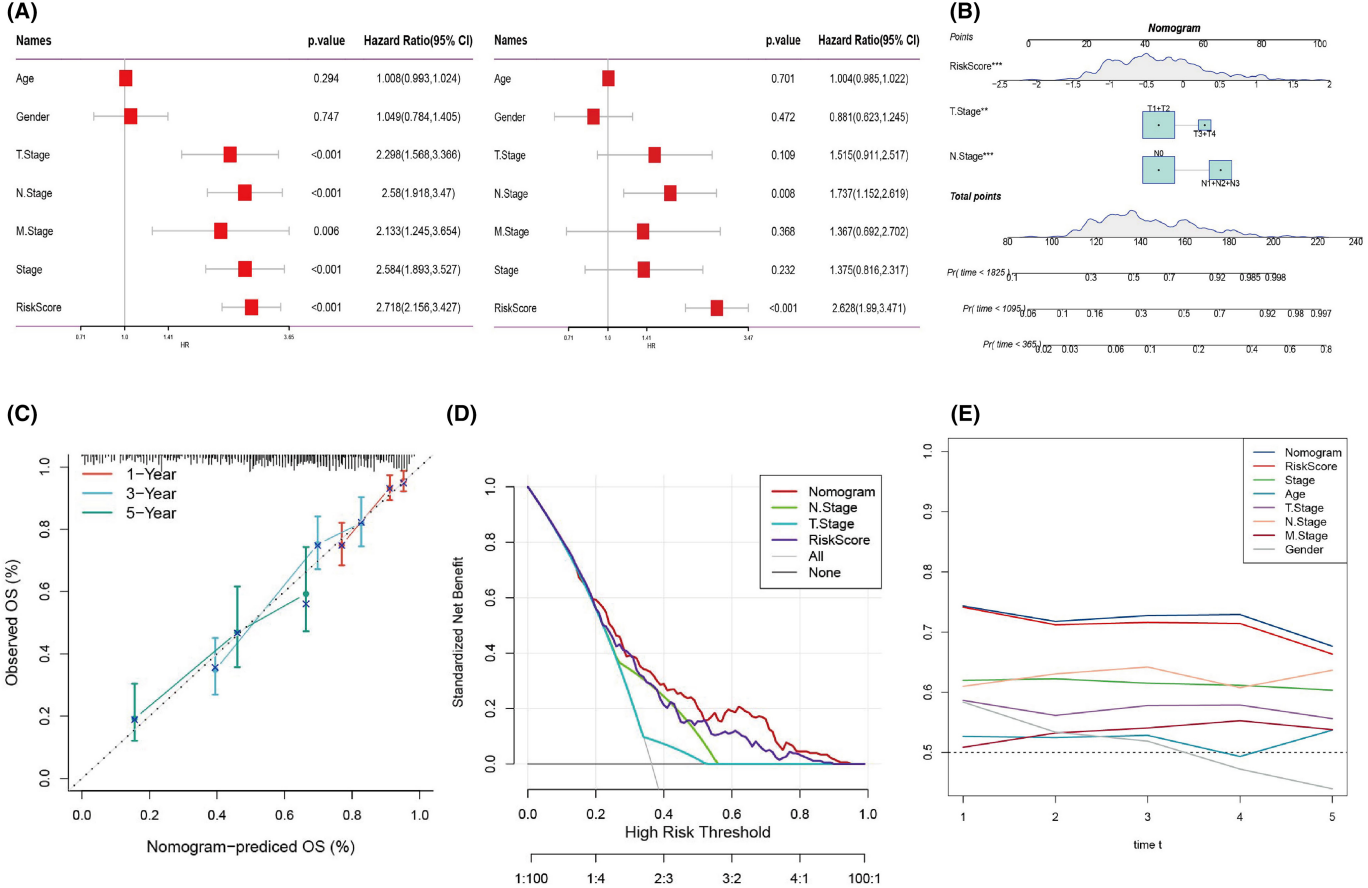
Establishment of the bilirubin metabolism‐associated genes (BMAG) nomogram in predicting lung adenocarcinoma (LUAD) prognosis. A: Univariate and multivariate analyses of BMAG score and clinicopathological features. B: Nomogram model including the risk score and stage. C: 1‐, 3‐ and 5‐year‐calibration curve nomogram. D: DCA plot of BMAG nomograph. E: Time‐ROC plot of LUAD clinicopathological features and BMAG nomogram.

Univariate and multivariate Cox regression analyses were performed to determine the predictive accuracy of the BMAG‐prognosis model by incorporating clinicopathological features. The risk signature for BMAG was the most crucial independent prognostic factor for LUAD (Figure 3A). As a result, a nomogram that combined stage and BMAG score was created (Figure 3B). The calibration plot verified that the BMAG nomogram was highly effective in predicting actual survival outcomes (Figure 3C). The discriminatory capacity of the BMAG nomogram for identifying high‐risk patients was superior (Figure 3D). Time‐ROC analysis demonstrated that the BMAG score and the nomogram outperformed other indicators within the TCGA cohort (Figure 3E).

### 3.3 Bilirubin metabolism‐associated gene‐prognostic signature predicted immune therapy response

**FIGURE 4:**
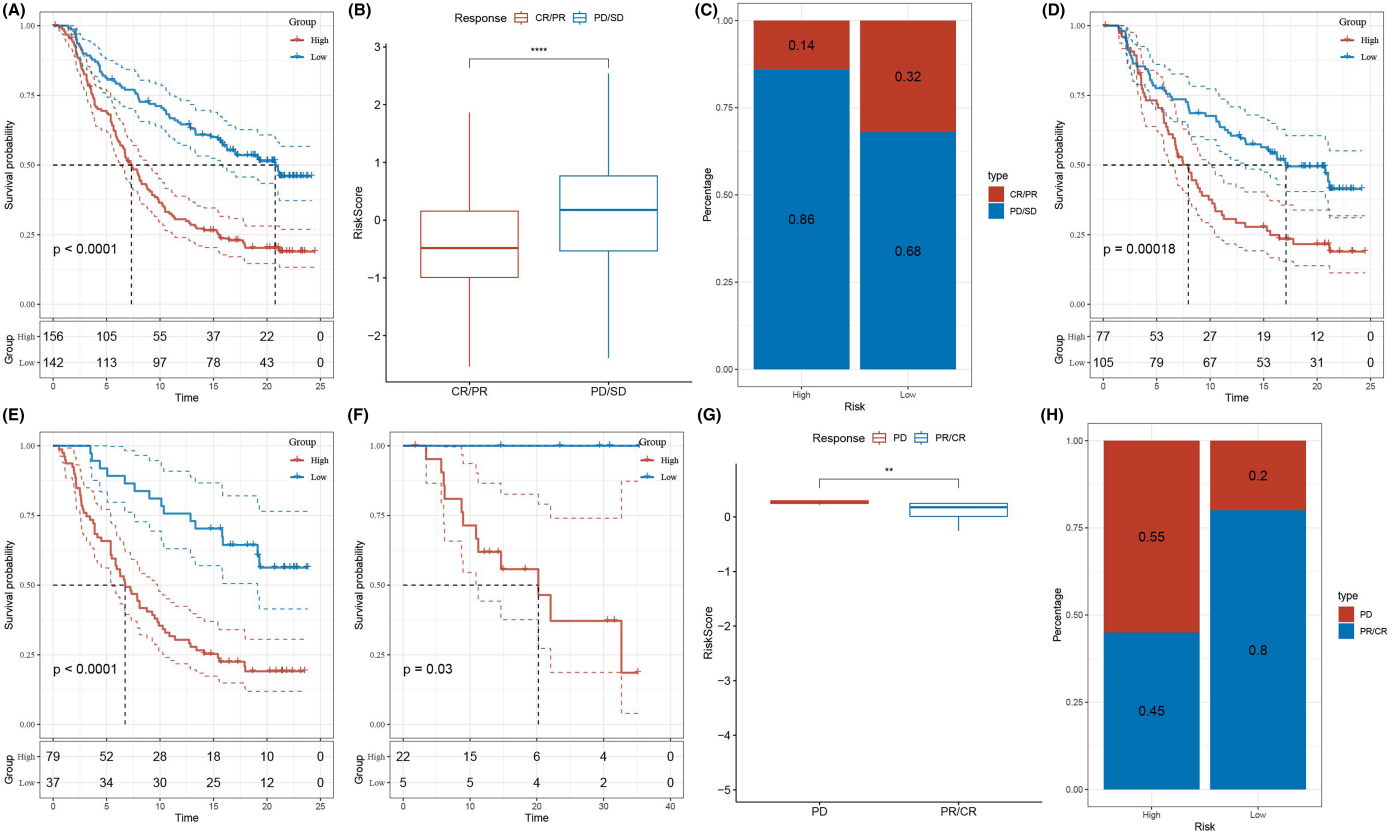
The therapeutic efficacy of the bilirubin metabolism‐associated genes (BMAG) risk model to immune checkpoint blockade (ICB) in the IMvigor210 cohort. A: Differences in the survival of two risk groups in The Cancer Genome Atlas‐lung adenocarcinoma (TCGA‐LUAD) cohort. B: BMAG risk scores among different ICB efficacy groups. C: Percentages of ICB response in the two groups. D: Survival curves of two risk groups in the IMvigor210 cohort. E,F: Survival curves of two risk groups in patients of I + II stage and III + IV. G: BMAG risk scores among different ICB efficacy groups. H: Percentages of ICB response among two risk groups.

Employing the BMAGs prognostic model, we found significantly diminished overall survival of the high‐risk group compared to the low‐risk group (Figure 4A). ICB therapy has gained prominence as an anti‐cancer strategy, offering favourable synergistic survival.^19^ Consequently, we evaluated the prognostic significance of BMAG risk signature concerning ICB. The IMvigor210 cohort, comprising 348 patients, exhibited varying responses to anti‐PD‐L1 receptor inhibitors and included complete response (CR), partial response (PR), stable disease (SD) and progressive disease (PD).^20^ BMAG scores of patients with SD/PD were higher than those with CR/PR (Figure 4B). The proportion of SD/PD cases in the high‐risk group exceeded that of the low‐risk group (Figure 4C). Notably, compared to high‐risk patients, low‐risk patients experienced substantial clinical benefits and significantly extended overall survival in the IMvigor210 cohort (Figure 4D). In particular, there was a significant difference in survival among Stage I+II patients between the distinct risk groups (Figure 4E), while no such difference was observed for Stage III+IV patients (Figure 4F). Collectively, these data suggest that our BMAG scoring system possesses greater sensitivity for early‐stage patients. Compared to the PR/CR group, the BMAG risk score of the PD group was significantly increased (Figure 4G). Concurrently, the proportion of PD cases in the high‐risk group exceeded that of the low‐risk group (Figure 4H).

### 3.4 Somatic mutation analysis of bilirubin metabolism‐associated gene‐prognostic signature

**FIGURE 5:**
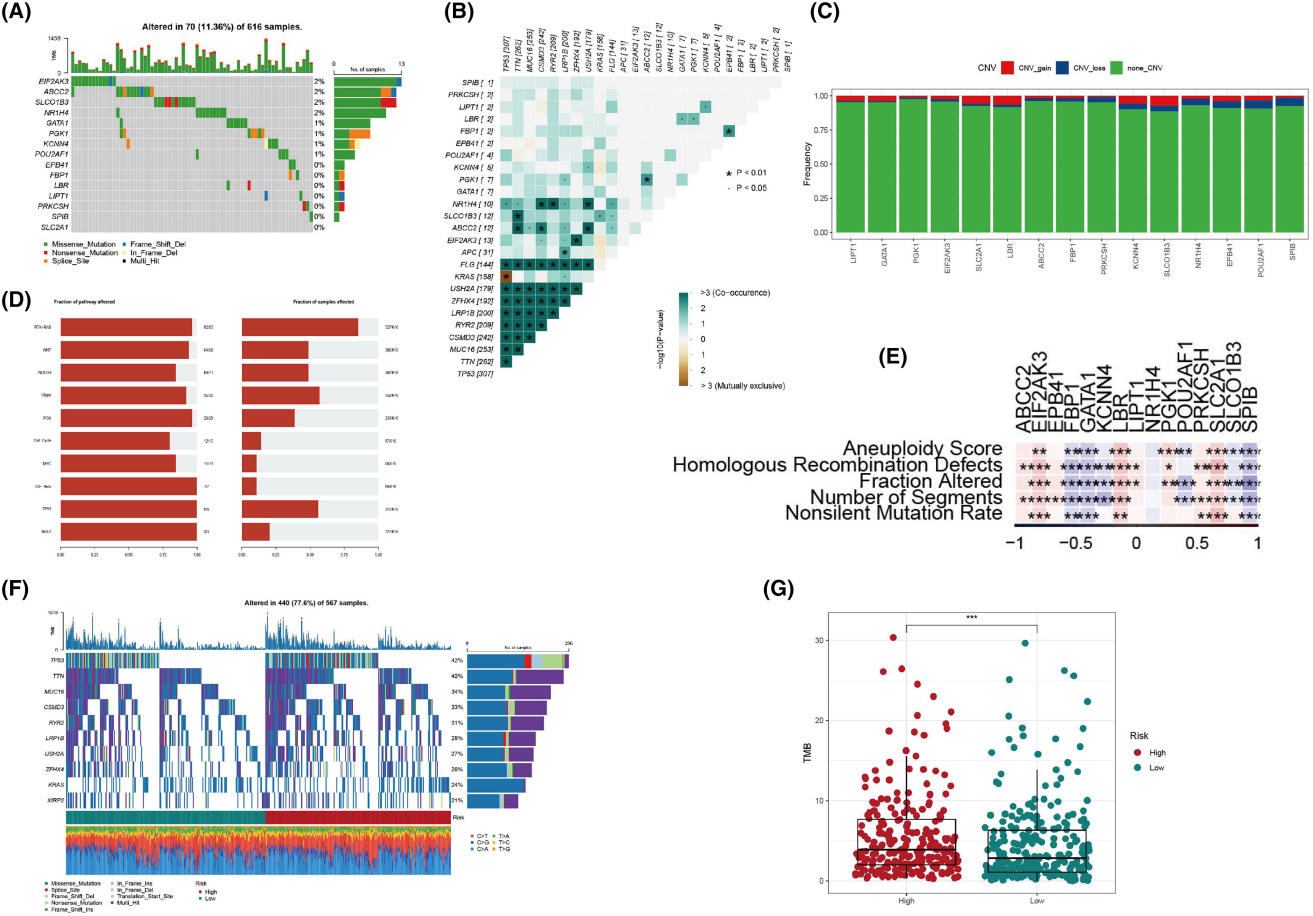
Somatic mutation analysis. A: Oncoprint visualization of the top 15 most frequently mutated genes. B: Co‐occurrence probability analysis of mutated genes. C: Copy number variation mutations (gain, loss, none) of the top 15 mutated genes. D: The mutation frequencies of nine major oncogenic pathways in lung adenocarcinoma (LUAD). E: Correlation heatmap of 15 bilirubin metabolism‐associated genes (BMAGs) with Aneuploidy Score, Homologous Recombination Defects, Fraction Altered, Number of Segments and Nonsilent Mutation Rate. F: Oncoprint visualization of the top 15 most frequently mutated genes in two risk groups G: Tumour Mutation Burden score between two risk groups.

**FIGURE 6:**
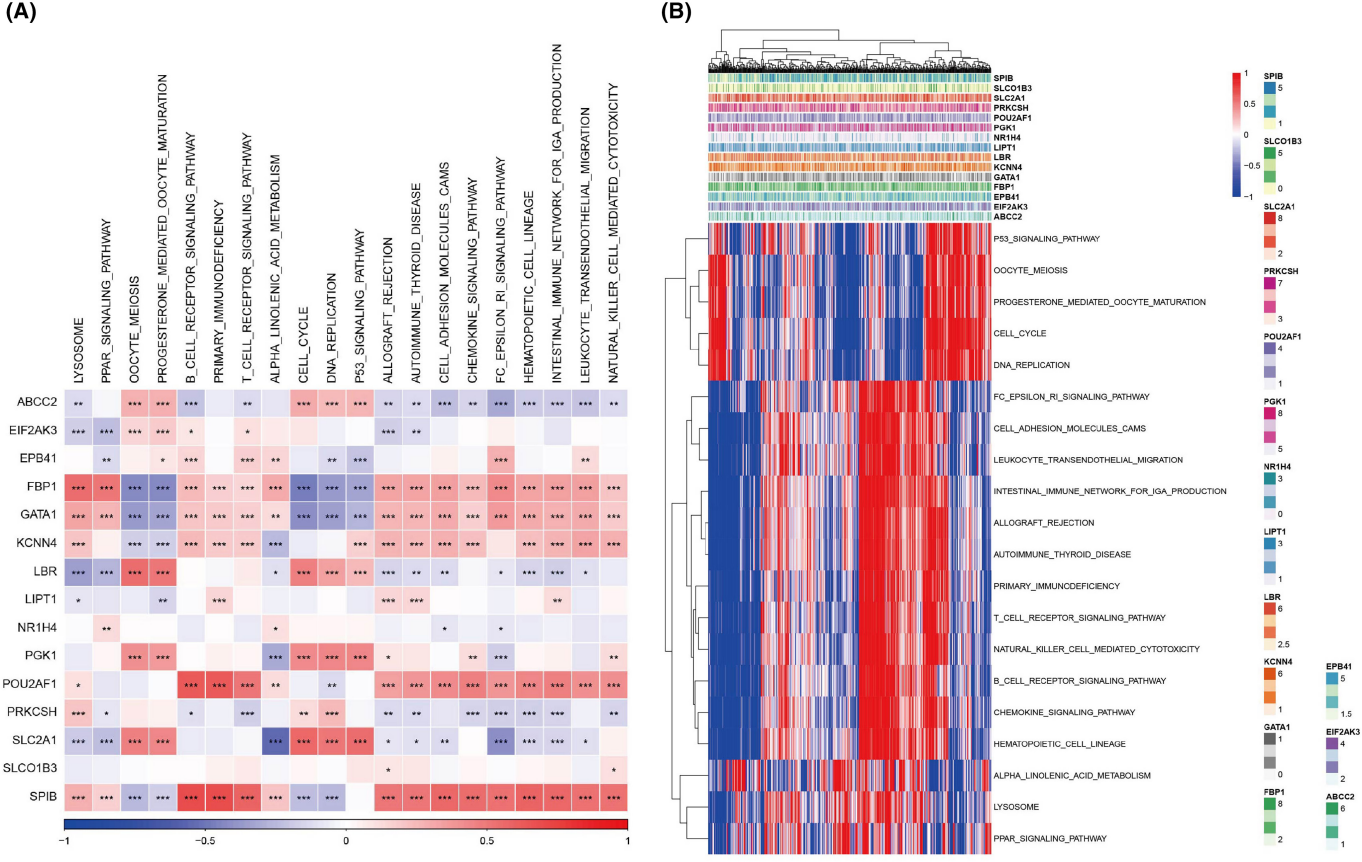
Identification of pathways that 15 bilirubin metabolism‐associated genes (BMAGs) were involved in A: BMAGs‐pathway correlation heatmap. B: Enrichment score heatmap of significant signalling pathways.

We investigated the overall somatic mutation landscape of patients with LUAD and the top five missense mutation mRNAs identified were EIF2AK3, ABCC2, SLCO1B3, NR1H4 and GATA1 (Figure 5A). The co‐occurrence probability of mutated genes identified a strong probability of co‐occurrence of mutation in FLG, KRAS, USH2A, ZFHX4, LRP1B, RYR2, CSMD3, MUC16, TTN and TP53 except for TP53 and KRAS (Figure 5B). Only very limited patients with LUAD had gain/loss of CNV (Figure 5C). Nine major oncogenic pathways, including RTK‐Ras, Wnt, Notch, Hippo, PI3K and cell cycle signalling pathways, were detected in LUAD samples (Figure 5D). Assessment of the relationships between BMAGs and various molecular signatures of LUAD revealed a significant negative correlation between FBP1, GATA1, KCNN4 and SPB, and the homologous recombination defects (HRD) activity (Figure 5E). On the other hand, EIF2AK3, LBR and SLC2A1 exhibited significantly positive correlations with HRD phenotypes. We explored the somatic mutation pattern between the two risk groups to gain a deeper understanding of the connections between prognostic‐related BMAGs and LUAD (Figure 5F). The risk group exhibited reduced TMB abundance (Figure 5G). In addition, cell cycle, cell migration and immune cell signalling pathways were found to be significantly associated with 15 BMAGs (Figure 6A,B).

### 3.5 Association analysis between bilirubin metabolism‐associated genes and immune cell

**FIGURE 7:**
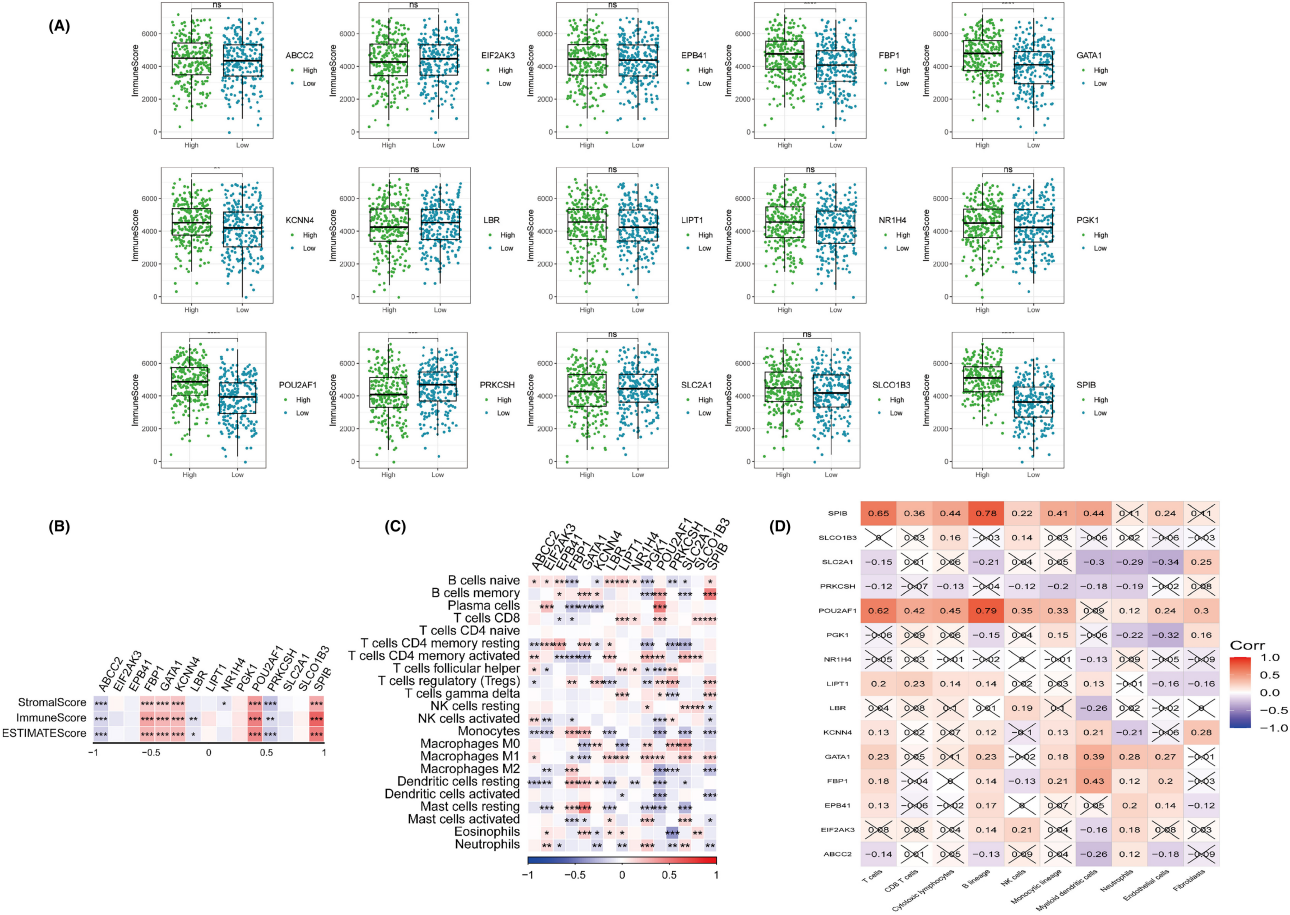
Association analysis between bilirubin metabolism‐associated genes (BMAGs) and immune cells. A: Immune store of 15 BMAGs in two risk groups. B: Immune score heatmap of 15 BMAGs in the two assessed risk groups. C: Heatmap of immune cell infiltration across 15 BMAGs in tumour samples. D: Correlation matrix of expression profiles of 15 BMAGs in various immune cell types.

The aforementioned GSEA revealed strong enrichment of immune cell‐associated signalling pathways with BMAGs, prompting us to delve into the relationship between immune cell infiltration and 15 BMAGs. We found the association of FBP1, GATA1, KCNN4, POU2AF1 and SPIB with heightened immune cell infiltration in patients of the high‐risk group, while ABCC2 and PRKCSH demonstrated notably lower levels of immune infiltration (Figure 7A,B). Furthermore, a positive association existed between SPIB and POU2AF1 with various immune cell types (Figure 7C,D).

### 3.6 Immune landscape and molecular expression of bilirubin metabolism‐associated genes signature

**FIGURE 8:**
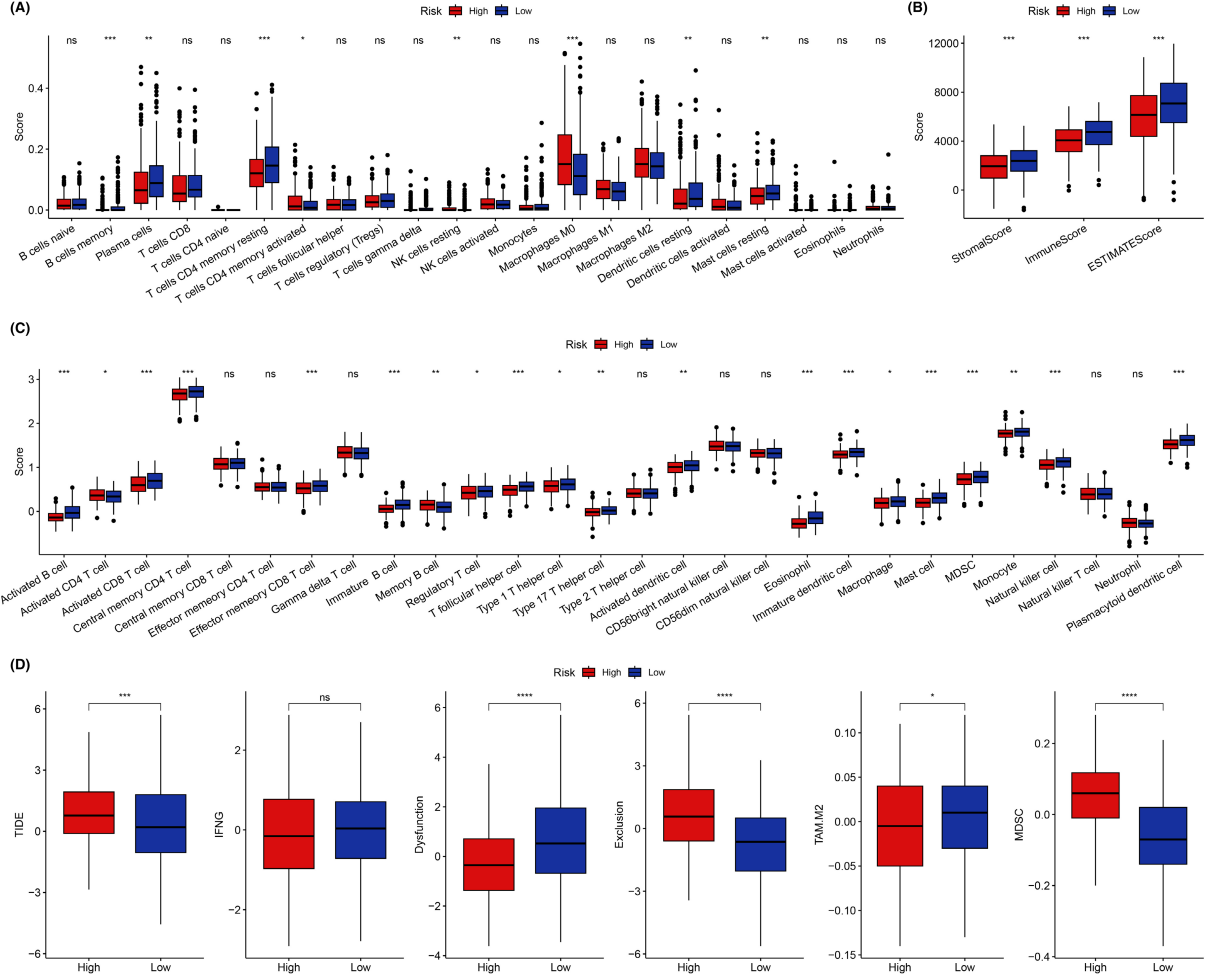
Immune cell infiltration between two (low and high) risk groups. A: Immune cell infiltration of various immune cell types in two groups. B: Immune score of two groups. C: Active immune cell infiltration of various types of immune cells in two groups. D: Tumour Immune Dysfunction and Exclusion (TIDE) score in two groups.

**FIGURE 9:**
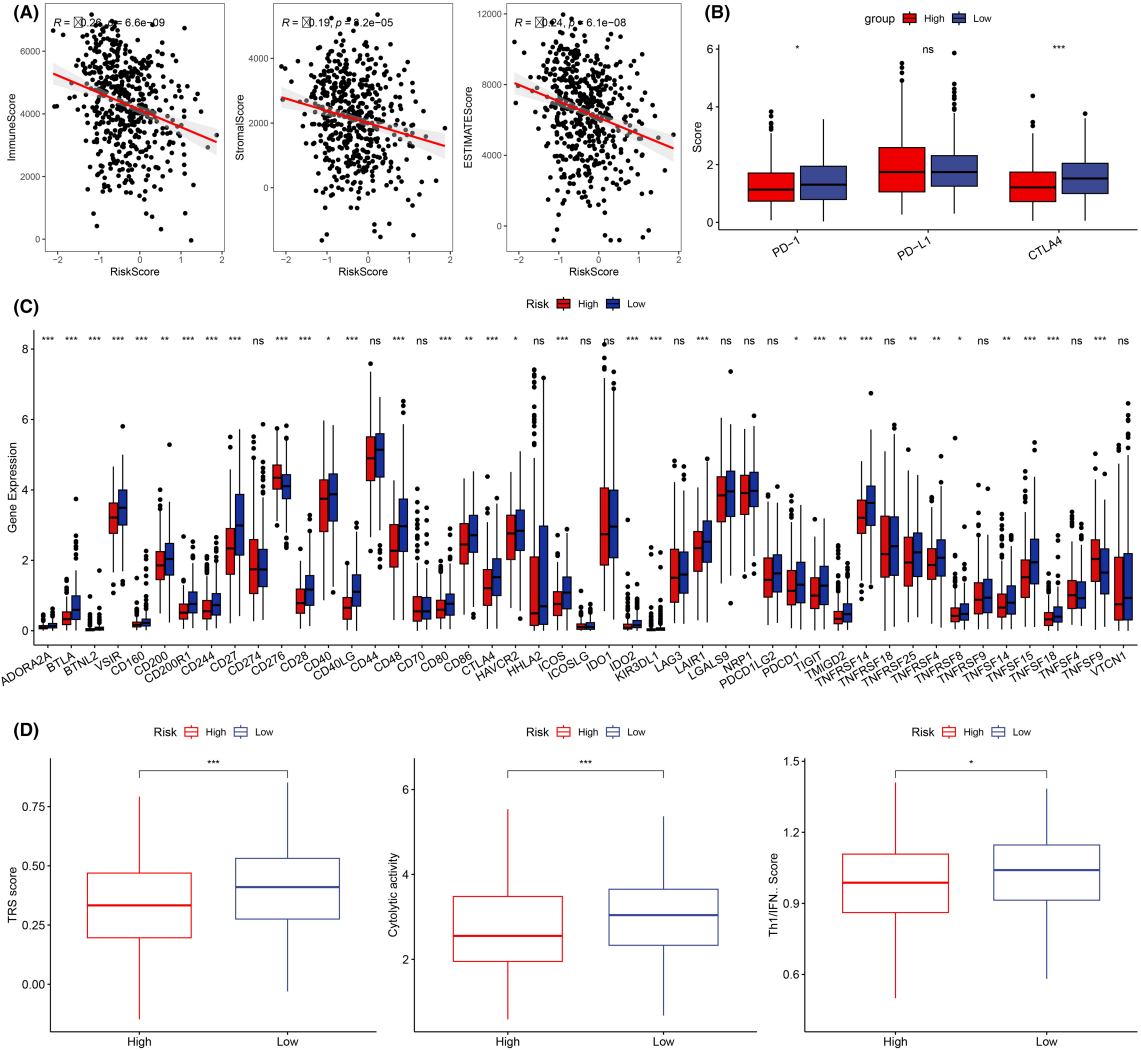
Immune checkpoint molecule (ICM) expression in two (low and high) risk groups. A: Correlation between bilirubin metabolism‐associated genes (BMAG) risk score and immune score. B: Expression of PD‐1 and CTLA‐4 in two risk groups. C: 47 Expression of ICM in two risk groups. D: tumour rejection signature (TRS), cytolytic activity (CYT) and Th1/interferon‐gamma (Th1/IFN) scores of two risk groups.

Next, we examined the immune cell infiltration differences and expression of immune checkpoint molecules (ICMs) across both risk groups. We employed ssGSEA and discovered that the low BMAGs risk group exhibited a comparatively enhanced infiltration abundance of immune cell subpopulations, particularly active immune cell groups (Figure 8A,C). Furthermore, the low‐risk group had a lower TIDE score than that of the high‐risk group (Figure 8D). The immune score and stromal score were negatively associated with BMAG risk score (Figure 9A). Also, the two key ICM genes PD‐1 and CTLA‐4 were highly expressed in the low‐risk group (Figure 9B). Furthermore, among the 47 ICMs under consideration, the low BMAGs group displayed a significantly elevated relative expression for most of these 47 ICMs (Figure 9C). The immunotherapeutic potential of the high‐ and low‐risk groups was evaluated by comparing the expression of three immunotherapeutic predictive biomarkers: the chemokine‐related signature (CRS) score, CYT score and Th1/ IFN‐γ score (Figure 9D). These scores were enhanced in the low‐risk group. These findings consistently suggest that patients with LUAD having low BMAGs exhibit a greater potential to respond favourably to immunotherapy.

### 3.7 Identification of drug candidates for patients with high‐risk bilirubin metabolism‐associated genes

**FIGURE 10:**
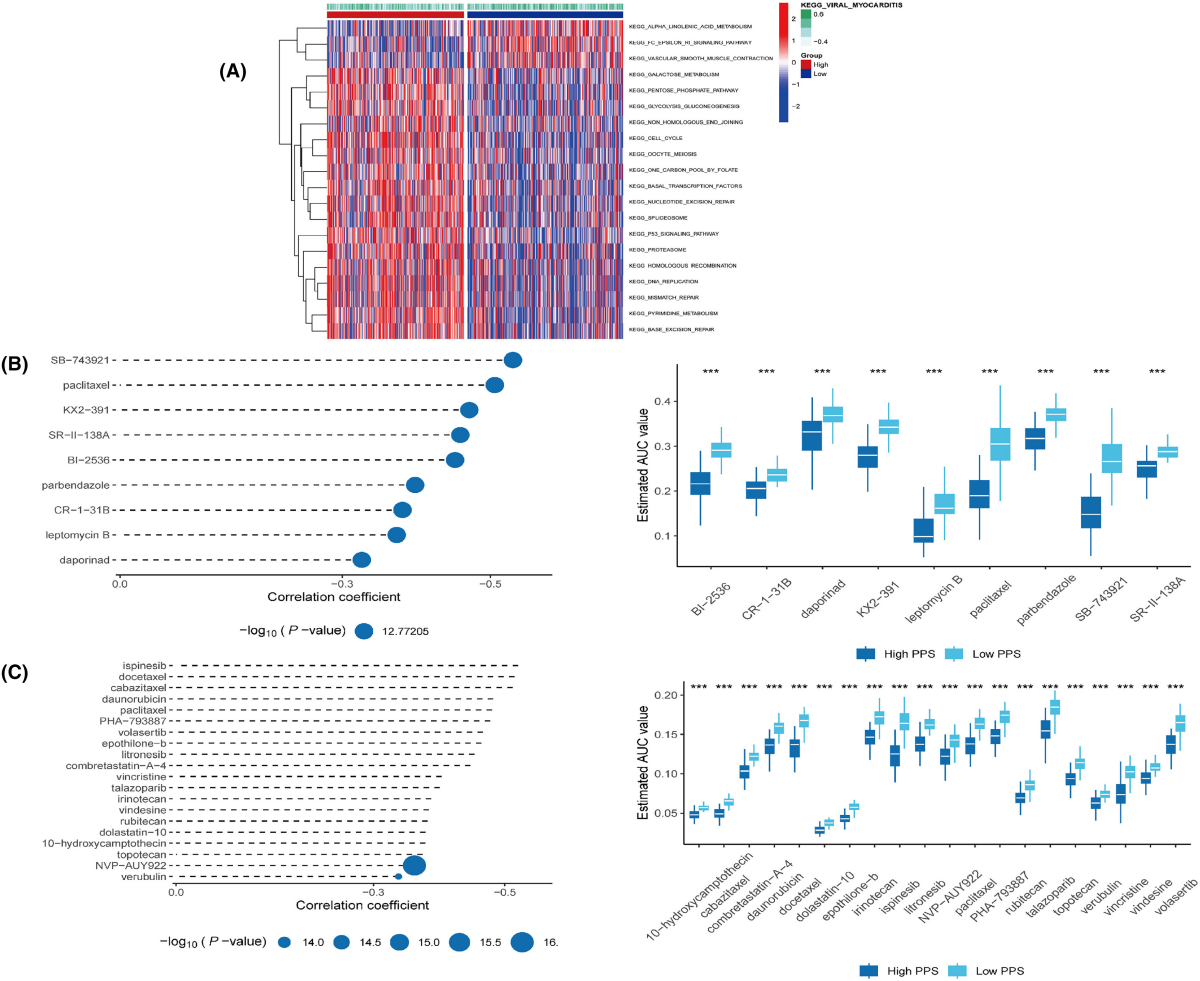
Identification of drug candidates for high bilirubin metabolism‐associated gene (BMAG) risk patients. A: Kyoto Encyclopedia of Genes and Genomes analysis between two risk groups. B‐C: Spearman's correlation analysis and drug response of 10 CTRP‐derived compounds (B) and 20 PRISM‐derived compounds (C) in two risk groups.

Kyoto Encyclopedia of Genes and Genomes analysis suggested the involvement of mainly linolenic acid metabolism, epsilon RI signalling pathway and vascular smooth muscle contraction signalling in the low‐risk group (Figure 10A). As the low‐risk group benefited from the immune therapy, we then applied two distinct strategies to determine candidate agents exhibiting increased drug sensitivity in high‐risk groups (for the analyses, CTRP and PRISM‐derived drug response data were used). The CTRP‐derived analysis identified nine therapeutic agents (BI‐2536, CR1‐31B, daporinad, KX2‐391, leptomycin, paclitaxel, parbendazole, SB‐743921 and SR‐II‐138A) (Figure 10B) and PRISM‐derived compounds analysis identified 20 therapeutic drugs (Figure 10C).

### 3.8 Fructose bisphosphate 1 plays an anti‐carcinogenic role in vitro

**FIGURE 11:**
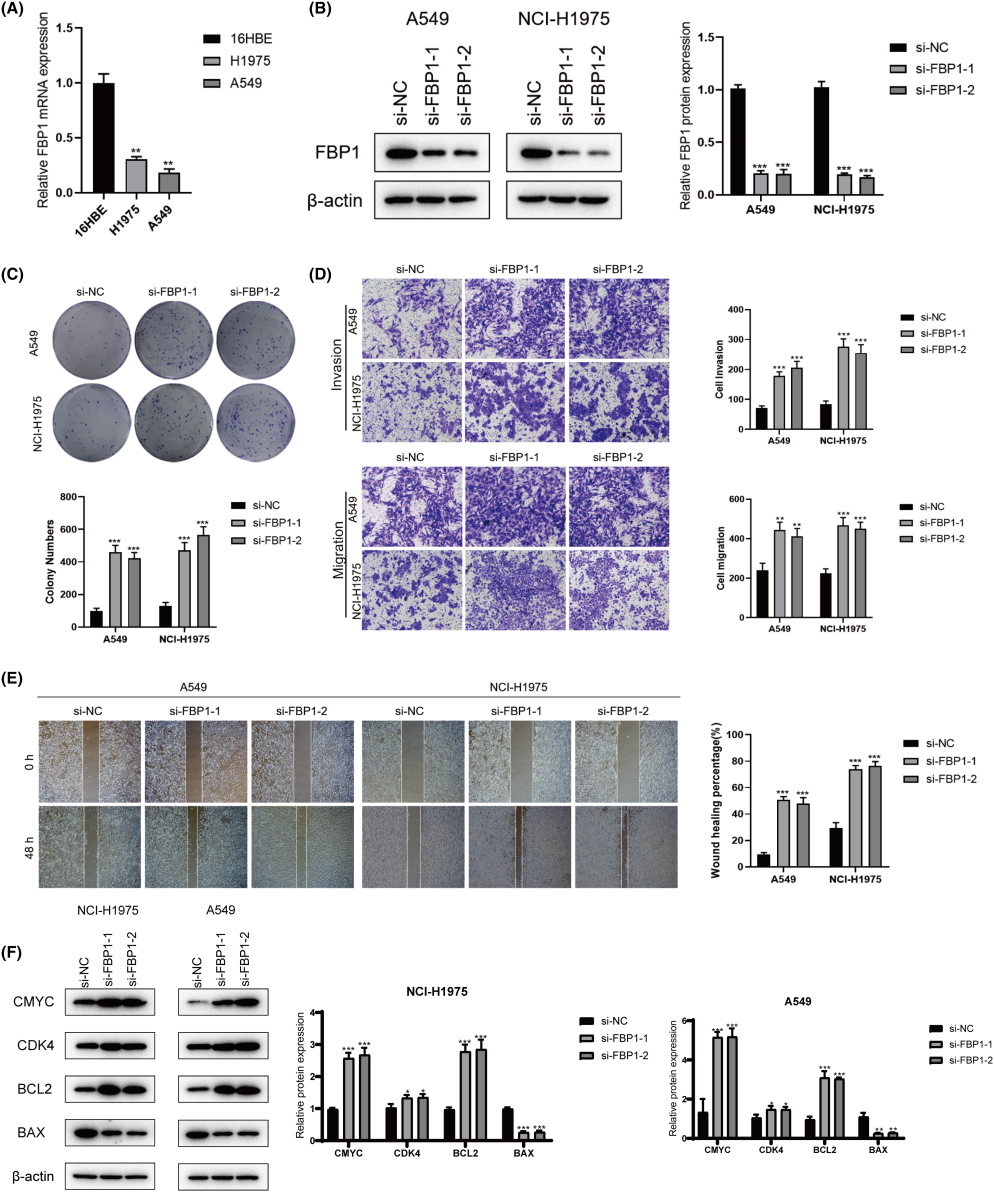
Knockdown of fructose bisphosphate 1 (FBP1) promotes lung cancer LC progression. A: Relative FBP1 expression in NCI‐H1975, A549 and 16HBE cells was detected by quantitative real‐time polymerase chain reaction. B: Western blot analysis of the relative FBP1 expression after FBP1 knockdown in A549 and NCI‐H1975 cells. C: Effect of FBP1 silencing on the proliferation in A549 and NCI‐H1975 cells was determined through by colony formation assays. D: Transwell evaluated the migration and invasion ability of cells after FBP1 knockdown. E: The changes in cell migration ability were determined through wound healing assays after FBP1 knockdown. F: Changes in the levels of key apoptotic proteins (bcl‐2, bax) and key proteins in the cell cycle (C‐Myc, CDK4) were detected by western blotting in A549 and NCI‐H1975 cells after FBP1 knockdown. *p < 0.05, **p < 0.01, ***p < 0.001.

We explored the relationship between FBP1 and various clinical features and found that its expression was high in the early stages of LUAD (File S2), while low FBP1 expression predicted poor survival (File S3). We also analysed the expression of FBP1 in pan‐cancer and in the single‐cell transcriptome. We found that FBP1 was highly expressed in most tumours except LUAD (File S4), while FBP1 was also highly expressed in macrophages, which can be used as a marker gene for macrophages (File S5). FBP1 expression was significantly decreased in NCI‐H1975 and A549 cells (Figure 11A). To define the function of FBP1 in LC cells, we knocked down the expression of FBP1 in NCI‐H1975 and A549 cells employing two specific siRNAs (si‐FBP1#1, si‐FBP1#2). The efficiency of transfection was verified by western blotting, and the expression of FBP1 was obviously diminished in cells after transfection of si‐FBP1#1 and si‐FBP1#2 (Figure 11B). Colony formation assays indicated that the clonogenicity of A549 and NCI‐H1975 cells was significantly promoted after FBP1 downregulation (Figure 11C). In addition, the transwell assays showed that migration and invasion of A549 and NCI‐H1975 cells were enhanced after FBP1 silencing (Figure 11D). In the wound healing assay, there was a distinct increase in relative healing areas in si‐FBP1 groups (Figure 11E). In the FBP1 knockdown group, the levels of the anti‐apoptotic protein BCL‐2 markedly increased while that of BAX decreased in NCI‐H1975 and A549 cells (Figure 11F). In the FBP1 knockdown group, the levels of the anti‐apoptotic protein bcl‐2 markedly decreased while that of bax increased in A549 and NCI‐H1975 cells (Figure 11F), demonstrating that FBP1 plays a tumour‐suppressive role in LC. Collectively, these results illuminate the biological role of reduced FBP1 expression in the critical characteristics of LC cells.

## 4 DISCUSSION

Bilirubin, a byproduct of haem catabolism, has been recently found as a potential modulator of cancer progression and immune response. The levels of circulating bilirubin are associated with the prognosis of colorectal cancer (CRC), and the higher level of UGT1A1 single nucleotide polymorphism (SNP) predicts a 7% increase in CRC risk.^21^ The validity and reliability of albumin‐bilirubin (ALBI) grading of liver function has made a significant difference in HCC prognosis and decision‐making.^22^ For example, when patients with HCC undergoing transarterial chemoembolization (TACE) therapy are diagnosed with a high ALBI grade in the pretreatment stage, they experience a poorer prognosis,^23^ proving ALBI grade as a useful tool for clinical prognostication and patient selection for TACE.

In our study, we performed comprehensive bioinformatics analyses to elucidate the role of BMAGs in the prognosis of LUAD and response to immunotherapy. We could identify a prognostic signature of 15 BMAGs that reliably predicts LUAD outcomes, which was corroborated across five independent GEO datasets. Notably, the linkage of ABCC2 to the prognosis of LUAD has been established, forming the basis of a prognostic nomogram.^24^ Moreover, diminished expression of EIF2AK3 in LUAD cells has been implicated in prognostic outcomes.^25^ The BMAG‐prognostic signature was shown to be an independent factor for prognosis when combined with clinicopathological features, outperforming other indicators. The nomogram we prepared, which incorporated the BMAG risk score and tumour stage, demonstrated high predictive accuracy and discriminatory capacity in the identification of high‐risk patients. This finding suggests that the prognostic signature could potentially serve as a valuable tool to guide treatment decisions and follow‐up strategies for patients with LUAD. However, further validation in prospective studies is warranted to confirm the clinical utility of this signature.

Further inquiries into pivotal BMAGs uncovered their regulatory roles in LUAD advancement. FBP1 plays a pivotal role in the metabolic reprogramming of LUAD cells. FBP1, as a rate‐limiting enzyme in gluconeogenesis, catalyses the conversion of fructose‐1,6‐bisphosphate to fructose‐6‐phosphate and is a negative regulator of aerobic glycolysis in LUAD cells.^26^ It is commonly downregulated in a variety of solid tumours and has been implicated as a tumour suppressor; its reduced expression is linked to the Warburg effect, ultimately hindering cancer cell proliferation.^27^ The reduced expression of FBP1 is associated with tumorigenesis and adverse prognosis in several cancers, including LUAD.^27^, ^28^ FBP1 curtails PD‐L1 expression by interacting with the STAT3 pathway, and its absence contributes to the immune evasion of malignant cells.^29^ Furthermore, hypoxia stimulates hypoxia‐inducible factor 1α (HIF1α)‐mediated GBE1 elevation, leading to the suppression of FBP1 levels of promoter methylation through NF‐κB signalling in LUAD cells.^30^ Suppression of FBP1 upregulated HIF1α, instigating a shift to anaerobic glycolysis and enhanced glucose uptake, thereby facilitating the progression of LUAD.^30^ In our study, we confirmed that FBP1 impedes LUAD cell proliferation and metastasis in vitro and is associated with cell cycle regulation and apoptosis, determined by subsequent functional assays. Therefore, augmenting FBP1 expression can be a potential strategy to counteract LUAD progression. PGK1 serves as a core gene in tumour metabolism, particularly glycolysis, and plays a determining role in evaluating immune therapy response, as indicated by the over‐expression of PGK1 in immunosuppressive cells and significant correlations to the expression of ICMs.^31^

We explored the somatic mutation landscape in LUAD and delineated key molecular alterations associated with the BMAG‐prognostic signature, distinguishing two risk groups with unique molecular profiles. Notably, EIF2AK3 emerged as the most frequently mutated mRNA, characterized predominantly by missense mutations. Furthermore, our analysis uncovered dysregulation in several oncogenic pathways, including RTK‐RAS, PI3K and cell cycle signalling. Intriguingly, while RAS signalling through the PI3K/AKT pathway typically promotes cell survival, it can paradoxically also trigger apoptosis.^32^, ^33^ The co‐occurrence of the mutations landscape highlighted a sophisticated network of genetic interactions potentially pivotal in LUAD development. It has been demonstrated that the activation of the EIF2AK3‐EIF2S1 pathway by uric acid (UA) exhibited cytoprotective properties, shielding cells from UA‐induced apoptosis.^34^ This protective mechanism involved EIF2AK3‐driven upregulation of MCL1 through ATF4 transcriptional activation. In MCF‐7 human breast cancer cells, sublethal UA exposure triggers autophagy‐associated ER stress, attenuating UA‐induced apoptosis via the EIF2AK3‐mediated increase of MCL1.^35^ Additionally, genes including EIF2AK3, LBR and SLC2A1 demonstrated significant correlations with HRD, intimating a potential association with the DNA damage response pathway in LUAD (Figure 5E). This observation, alongside other studies highlighting HRD and chromosomal instability's roles in LUAD progression,^36^, ^37^ underscores the complex molecular underpinnings of this cancer type, offering novel insights for targeted therapeutic strategies.

Recent progress in oncology has highlighted immunotherapies, such as ICB and adoptive cell therapies, as critical modalities. Our study reflected this paradigm shift and explored the impact of BMAGs on the immunotherapeutic response in LUAD. Our BMAG‐based prognostic signature is a robust predictor of ICB therapy responsiveness. The use of ICB has shown significant clinical success in immunotherapy targeting checkpoint proteins, such as PD‐L1 on tumour cells and PD‐1 on T cells, to enhance antitumor immunity.^18^ Blocking the PD‐L1 and PD‐1 interaction with immune checkpoint inhibitors, such as anti‐PD‐1 and CTLA‐4, allows T cells to recognize and effectively eliminate tumour cells.^19^, ^20^ Here, we discovered that patients in the low‐risk group derived significant clinical benefits and extended overall survival than those in the high‐risk group, particularly among early‐stage patients. This finding highlights the potential value of the BMAG risk score in identifying patients more likely to respond favourably to anti‐PD‐1 and CTLA‐4 therapy, which could ultimately improve treatment outcomes and minimize unnecessary side effects.

The TME plays a crucial role in cancer development and progression. TME comprises various elements, including extracellular matrix, cancer‐associated fibroblasts, vascular epithelial cells and infiltrating immune cells.^38^, ^39^ Immune cell infiltration in solid tumours, particularly in LUAD, has been identified as a key factor in autogenous or engineered ‘off‐the‐shelf’ cell product therapy.^40^ Among the multiple infiltrating immune cells, T‐cell subsets, including CD4+ T cells and Tregs, as well as polarized macrophages (M1 and M2 subtypes), have been shown to reshape immune cell therapy response.^41^, ^42^ However, the immune infiltration pattern in most malignancies cannot be characterized by a single‐cell type alone. Therefore, constructing a gene signature representing the overall immune cell infiltration status is critical for evaluating the effectiveness of immune‐related therapies in LUAD. Our immune infiltration analysis revealed that the low BMAG risk group had increased immune cell infiltration, particularly in active immune cell subpopulations, and higher expression levels of many ICMs. Additionally, the low‐risk group had elevated TRS, CYT and Th1/IFN‐γ scores, further supporting the notion that these patients are more likely to respond favourably to immune cell therapy.

Finally, we identified potential therapeutic agents exhibiting increased drug sensitivity, specifically in the high‐BMAG risk score group. Nine therapeutic agents (BI‐2536, CR1‐31B, daporinad, KX2‐391, leptomycin, paclitaxel, parbendazole, SB‐743921 and SR‐II‐138A) and 20 therapeutic drugs from the CTRP and PRISM data were identified. Our dual‐pronged approach resulted in identifying several candidate agents suitable for high BMAGs risk signature, which warrant further in vitro and in vivo investigation to validate their therapeutic potential in LUAD patients.

## 5 CONCLUSION

In conclusion, we generated a BMAG‐based prognosis signature that predicts the prognosis and immunotherapy response of LUAD patients accurately. The low‐risk group benefits more from immunotherapy, evidenced by increased immune cell infiltration, higher immune checkpoint expression and enrichment of immune‐related pathways. Our research elucidates the pivotal roles of the BMAG molecule within the context of LUAD, establishing a critical foundation for the development of BMAG‐centric personalized therapeutic strategies. However, the validation of these findings across a broader sample set remains an imperative next step.

## AUTHOR CONTRIBUTIONS

**Tonghai Huang:** Conceptualization (equal); data curation (equal). **Kangqi Ren:** Conceptualization (equal); data curation (equal). **Xiean Ling:** Conceptualization (equal); data curation (equal). **Lin Chen:** Conceptualization (equal); data curation (equal). **Zeyao Li:** Conceptualization (equal); data curation (equal).

## FUNDING INFORMATION

This study was supported by Natural Science Foundation of Shenzhen (NO.JCYJ20190806154601642) and Natural Science Foundation of Shenzhen (NO. JCYJ20230807112401002).

## CONFLICT OF INTEREST STATEMENT

The authors have no conflicts of interest to disclose.

## CONSENT FOR PUBLICATION

Not applicable.

## Supporting information


File S1.

File S2.

File S3.

File S4.

File S5.

Data S1.

## Data Availability

The data supporting the study findings are available from the corresponding author upon reasonable request.
