# Supplementary material for: Prognostic and immunotherapeutic implications of bilirubin metabolism‐associated genes in lung adenocarcinoma
Source: J Cell Mol Med. 2024 May 2;28(9):e18346. doi: 10.1111/jcmm.18346 (PMC11063731; doi:10.1111/jcmm.18346)
Supplement: Supplementary file 6 — Data S1. [file JCMM-28-e18346-s003.docx]

Supplementary file S1. Differential expression of 15 BMAGs in LUAD and normal

Supplementary file S2. Correlation between FBP1 and clinicopathological features

Supplementary file S3. Survival differences between high and low FBP1 expression groups.

Supplementary file S4. FBP1 expression in pan-cancers.

Supplementary file S5. Expression of FBP1 in single-cell sequencing.
